# A novel indole compound MA-35 attenuates renal fibrosis by inhibiting both TNF-α and TGF-β_1_ pathways

**DOI:** 10.1038/s41598-017-01702-7

**Published:** 2017-05-15

**Authors:** Hisato Shima, Kensuke Sasaki, Takehiro Suzuki, Chikahisa Mukawa, Ten Obara, Yuki Oba, Akihiro Matsuo, Takayasu Kobayashi, Eikan Mishima, Shun Watanabe, Yasutoshi Akiyama, Koichi Kikuchi, Tetsuro Matsuhashi, Yoshitsugu Oikawa, Fumika Nanto, Yukako Akiyama, Hsin-Jung Ho, Chitose Suzuki, Daisuke Saigusa, Atsushi Masamune, Yoshihisa Tomioka, Takao Masaki, Sadayoshi Ito, Ken-ichiro Hayashi, Takaaki Abe

**Affiliations:** 1 Division of Nephrology, Endocrinology, and Vascular Medicine, Tohoku University Graduate School of Medicine, Sendai, 980-8574 Japan; 2 Department of Nephrology, Hiroshima University Hospital, Hiroshima, 734-8551 Japan; 3 Department of Medical Science, Tohoku University Graduate School of Biomedical Engineering, Sendai, 980-8574 Japan; 4 Laboratory of Oncology, Pharmacy Practice and Sciences, Tohoku University Graduate School of Pharmaceutical Sciences, Sendai, 980-8578 Japan; 5 Department of Biology, Indiana University Purdue University Indianapolis, Indianapolis, IN 46202 USA; 6 Center for Gene Research, Tohoku University, Sendai, 980-8575 Japan; 7 Department of Clinical Biology and Hormonal Regulation, Tohoku University Graduate School of Medicine, Sendai, 980-8574 Japan; 8 Division of Pediatrics, Tohoku University Graduate School of Medicine, Sendai, Sendai 980-8574 Japan; 9 Department of Integrative Genomics, Tohoku Medical Megabank Organization, Tohoku University, Sendai, 980-8574 Japan; 10 Division of Gastroenterology, Tohoku University Graduate School of Medicine, Sendai, 980-8574 Japan; 11 Department of Biochemistry, Okayama University of Science, Okayama, 700-0005 Japan; 12 Indiana University School of Medicine, Indianapolis, IN, 46202 USA

## Abstract

Renal fibrosis is closely related to chronic inflammation and is under the control of epigenetic regulations. Because the signaling of transforming growth factor-β_1_ (TGF-β_1_) and tumor necrosis factor-α (TNF-α) play key roles in progression of renal fibrosis, dual blockade of TGF-β_1_ and TNF-α is desired as its therapeutic approach. Here we screened small molecules showing anti-TNF-α activity in the compound library of indole derivatives. 11 out of 41 indole derivatives inhibited the TNF-α effect. Among them, Mitochonic Acid 35 (MA-35), 5-(3, 5-dimethoxybenzyloxy)-3-indoleacetic acid, showed the potent effect. The anti-TNF-α activity was mediated by inhibiting IκB kinase phosphorylation, which attenuated the LPS/GaIN-induced hepatic inflammation in the mice. Additionally, MA-35 concurrently showed an anti-TGF-β_1_ effect by inhibiting Smad3 phosphorylation, resulting in the downregulation of TGF-β_1_-induced fibrotic gene expression. In unilateral ureter obstructed mouse kidney, which is a renal fibrosis model, MA-35 attenuated renal inflammation and fibrosis with the downregulation of inflammatory cytokines and fibrotic gene expressions. Furthermore, MA-35 inhibited TGF-β_1_-induced H3K4me1 histone modification of the fibrotic gene promoter, leading to a decrease in the fibrotic gene expression. MA-35 affects multiple signaling pathways involved in the fibrosis and may recover epigenetic modification; therefore, it could possibly be a novel therapeutic drug for fibrosis.

## Introduction

Fibrosis leads to the irreversible exacerbation of compromised function in various organs such as the liver, lung, heart and kidney^1^. In fibrotic diseases, the signaling of transforming growth factor-β_1_ (TGF-β_1_) plays a key role in its disease progression. TGF-β_1_ activates resident fibroblasts to turn into myofibroblasts, which produce excessive extracellular matrix (ECM)^1^. In addition to the TGF-β_1_ signaling, tumor necrosis factor-α (TNF-α) is an important pro-inflammatory mediator that aggravates renal fibrosis^2^, because renal fibrosis is preceded by chronic inflammation accompanied by complex inflammatory processes.

There is as yet no definitive drug for the treatment or prevention of renal fibrosis^3^, although several attempts to prevent fibrosis using anti-TNF-α agents have been undertaken. Anti-TNF-α biologics such as infliximab, adalimumab or etanercept have not been shown to be successful in the treatment of renal fibrosis. As in the case of such anti-TNF-α therapy, anti-TGF-β_1_ agents such as pirfenidone have not been shown to be successful in the treatment of renal fibrosis. Because TGF-β_1_ is one of the anti-inflammatory cytokines that suppresses inflammatory response, anti-TGF-β_1_ therapy alone may actually worsen the inflammatory response and thereby promote fibrosis^4^. Thus, inhibiting both the TNF-α and TGF-β_1_ signaling pathways may be a potential approach to anti-fibrotic treatment.

The pro-fibrotic effects of TGF-β_1_ are also controlled by epigenetic mechanisms^5, 6^. TGF-β_1_–induced SET domain-containing lysine methyltransferase 7/9 (SET7/9) promotes the methylation of histone H3 lysine 4 (H3K4) and increases pro-fibrotic gene expression, as we described previously. Thus, inhibition of SET7/9 could reverse histone modifications and it may prevent the progression of renal fibrosis.

In the present study, we focused on the development of a novel anti-fibrotic agent having both anti-TNF-α and anti-TGF-β_1_ activities. From the small molecule library of indole derivatives that we recently synthesized, we found out a novel indole derivative compound MA-35 that showed both anti-TNF-α and anti-TGF-β_1_ effect. MA-35 treatment attenuated the inflammatory response and renal fibrosis with epigenetic modification of SET7/9 *in vitro* and also in the animal model.

## Results

### Screening for anti-TNF-α compounds

**Figure 1:**
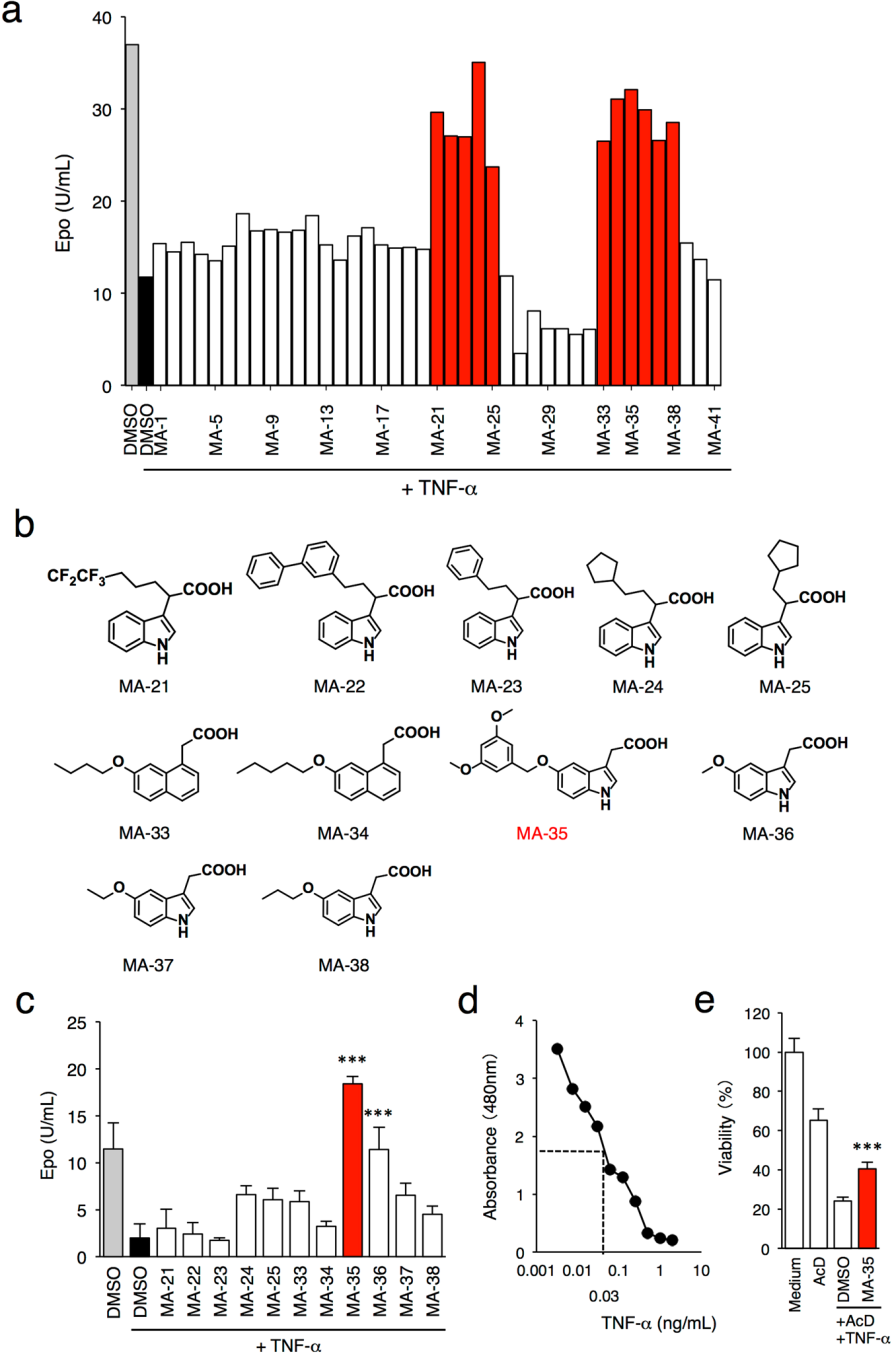
Indole derivatives exert an anti-TNF-α effect. (**a**) Initial screening of anti-TNF-α activity based on the recovery of Epo production suppressed by TNF-α exposure using Hep3B cells under a hypoxic condition in the presence of newly synthesized 41 indole derivatives. 11 derivatives rescued Epo production. (**b**) Chemical structure of the 11 indole derivatives that shows a putative anti-TNF-α activity. (**c**) Second screening of these 11 indole derivatives. MA-35 most significantly restored TNF-α-induced Epo suppression. (**d**) Cell viability of L929 cells under indicated concentration of TNF-α. The 50% survival value was 0.03 ng/dL. (**e**) Effect of MA-35 on TNF-α-induced L929 cell death. L929 cells were treated with TNF-α (0.03 ng/dL) and actinomycin D (10 μg/mL). The cell death rate was measured with MA-35 by cell viability assay. ***p < 0.001 v.s. DMSO+TNF-α.

To screen the compounds that have anti-TNF-α activity, we focused on the inhibitory effect of TNF-α on erythropoietin (Epo) production in liver-derived Hep3B cells. Epo production by the Hep3B cells increases under the hypoxic condition, and the increase is significantly suppressed by the exposure to TNF-α^7^. Recently, we constructed an in-house indole compound library and reported that some of compounds exerted a stimulatory effect on Epo production and increased the cellular ATP level in Epo-producing Hep3B cells^8, 9^. Then, we utilized this chemical library to screen compounds which have anti-TNF-α activity. As shown in Fig. 1a, exposure to TNF-α reduced the hypoxia-induced Epo production by 60% compared to the control. Under this condition, the cells were treated with 41 indole derivatives (MA-1 to MA-41) for 48 h. As a result, 11 compounds (MA-21, MA-22, MA-23, MA-24, MA-25, MA-33, MA-34, MA-35, MA-36, MA-37, and MA-38) restored the Epo reduction that suppressed by TNF-α to the normal level (Fig. 1a). The chemical structures of the compounds are shown in Fig. 1b. The secondary screening test also showed that TNF-α-mediated Epo reduction was significantly inhibited by MA-21, MA-24, MA-25, MA-33, MA-34, MA-35, MA-36, MA-37 and MA-38 (Fig. 1c), suggesting that these indole-derived compounds inhibited the effect of TNF-α. Among them, Mitochonic Acid 35 (MA-35), 5-(3, 5-dimethoxybenzyloxy)-3-indoleacetic acid, exhibited the most potent effect. In addition, treatment with MA-35 alone without TNF-α did not increase the Epo production in the Hep3B cells (Supplementary Fig. 1a). Similarly in the *in vitro* analysis, the intravenous injection of MA-35 into mice did not affect the serum Epo level (Supplementary Fig. 1b), suggesting that MA-35 is not an inducer of EPO production. These results indicate that MA-35 shows an anti-TNF-α activity that suppressed the Epo production.

To confirm the anti-TNF-α effect of MA-35, we next examined it using another *in vitro* system: a cytotoxicity assay using a TNF-α-sensitive L929 cells^10^. The cell viability of L929 cells was reduced by TNF-α in a dose-dependent manner and the apparent IC 50 was 0.03 ng/dL (Fig. 1d). L929 cells are also sensitized by actinomycin D^11^. The treatment of TNF-α with actinomycin D resulted in almost 75% cytotoxicity in L929 cells, whereas treatment with actinomycin D alone did not induce significant cell death. Under the condition, MA-35 treatment was able to inhibit TNF-α-induced cytotoxicity in L929 cells (Fig. 1e). These results demonstrated that the MA-35 possesses anti-TNF-α activity.

### MA-35 suppresses IKK phosphorylation and the positive feedback loop of TNF-α pathway

**Figure 2:**
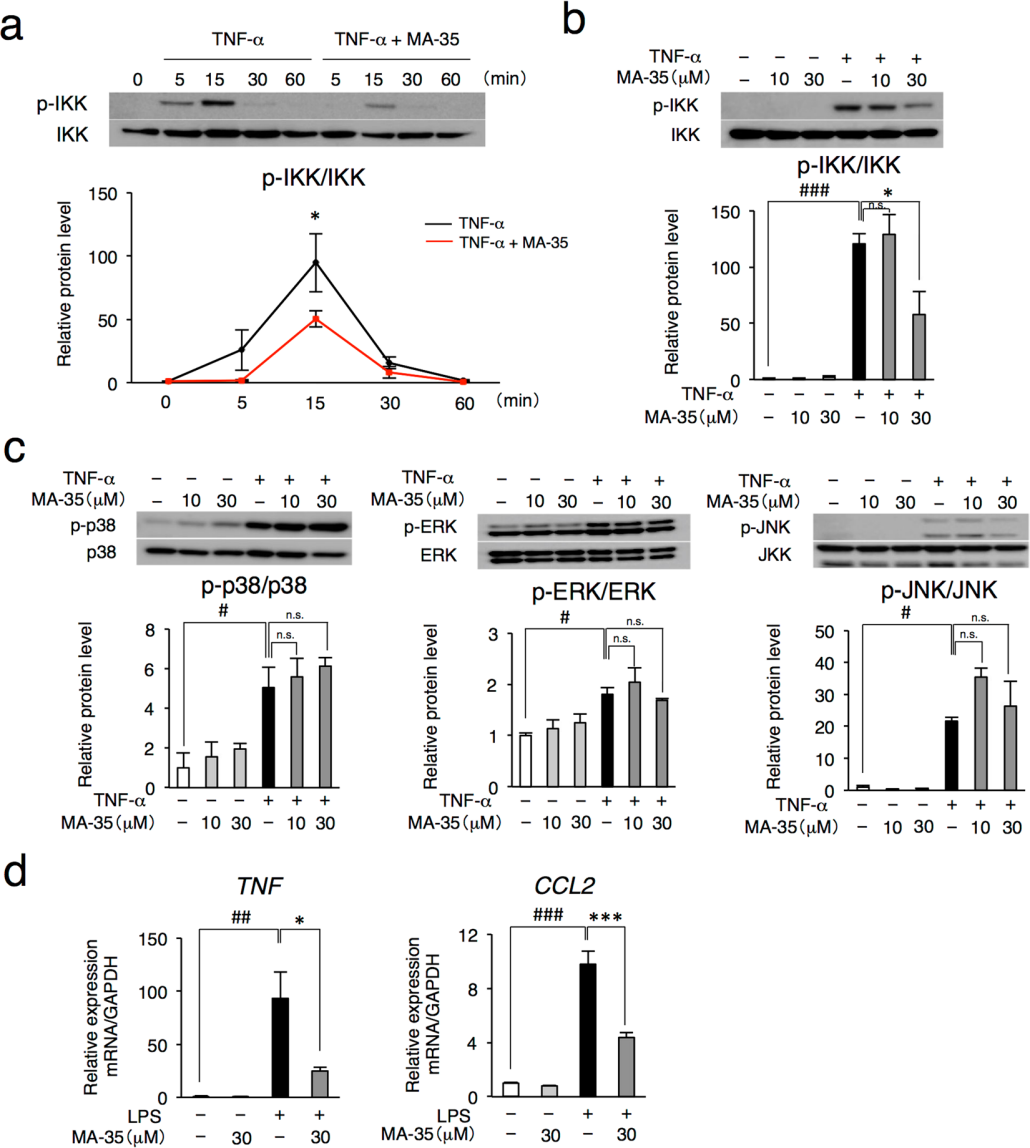
Effect of MA-35 on TNF-α-induced IKK phosphorylation and LPS-induced inflammatory cytokine expression in LX-2 cells. (**a**) Serum-starved LX-2 cells were preincubated for 60 min with or without MA-35 (30 μM) and then stimulated with TNF-α (10 ng/mL). Cell lysates were prepared at the indicated time points and analyzed by immunoblotting for p-IKK and IKK protein. The expression levels of p-IKK was quantified by densitometry and normalized with IKK. Full-length gel was shown in Supplementary Fig. 2. Serum-starved LX-2 cells were preincubated for 60 min with or without MA-35 (10 μM, 30 μM) and then stimulated with TNF-α (10 ng/mL). For (**b**) 15 min and (**c**) 30 min. The expression levels of (**b**) p-IKK, (**c**) p-p38, p-ERK and p-JNK were quantified by densitometry, and each was normalized with IKK, p38, ERK and JNK, respectively. Full-length gel was shown in Supplementary Fig. 4. Serum-starved LX-2 cells were preincubated for 60 min with or without MA-35 (30 μM) and then stimulated with LPS (100 ng/mL). (**d**) The mRNA level of *TNF* after 1 h LPS stimulation and *CCL2* after 3 h LPS stimulation were measured by real-time PCR. ^#^ *p* < 0.05, ^##^ *p* < 0.01, ^###^ *p* < 0.001 v.s. Control, *p < 0.05, ***p < 0.001 v.s. TNF-α or LPS.

To clarify the anti-TNF-α mechanism of MA-35, we examined the effect of MA-35 on the pathway of TNF-α signal transduction using human hepatic stellate cell (HSC) line LX-2, which is a representative model for examining TNF-α−^12^ and lipopolysaccharide (LPS) pathways^13^. Since TNF-α signaling pathway consists of phosphorylation of IκB kinase (IKK), p38, ERK and JNK, we examined the each phosphorylation level. The phosphorylation of IKK and p38 was increased 15 min after TNF-α stimulation, and the phosphorylation of ERK and JNK peaked at 30 min (Supplementary Fig. 2 and Supplementary Fig. 3). MA-35 significantly reduced the phosphorylation of IKK that induced by TNF-α (Fig. 2a and b). On the other hand, MA-35 did not influence on phosphorylation of p38, ERK and JNK induced by TNF-α (Fig. 2c and Supplementary Fig. 4). These data indicate that the anti-TNF-α effect of MA-35 is predominantly mediated by the inhibition of IKK phosphorylation in the TNF-α signaling pathway.

In the TNF-α inflammatory pathway, phosphorylated IKK degrades IκB, followed by the translocation of NF-κB p65/p50 into the nucleus, inducing the expression of pro-inflammatory genes such as TNF-α, MCP-1 and IL-6^14^. In addition, the induced-TNF-α further activates the IKK/NF-κB pathway and enhances the TNF-α expression, forming a positive feedback loop^15^. To examine whether MA-35 inhibits the downstream signaling of phosphorylated IKK and the positive feedback loop of TNF-α, we investigated the effect of MA-35 on the LPS/ Toll-like receptors 4 (TLR4) signaling pathway (Fig. 2d). LPS binds to TLR4 and then also phosphorylates IKK, resulting in the induction of inflammatory cytokines such as TNF-α and MCP-1. As shown in Fig. 2d, LPS treatment enhanced the mRNA expression of TNF-α and MCP-1 in the LX-2 cells. In this condition, MA-35 significantly decreased the TNF-α and MCP-1 mRNA levels induced by LPS (Fig. 2d). These findings suggest MA-35 suppressed inflammatory gene expressions induced by LPS, which would result from the inhibiting effect of MA-35 on the IKK phosphorylation and TNF-α positive feedback loop.

### MA-35 attenuates LPS/D-galactosamine-induced acute hepatitis by suppressing TNF-α

**Figure 3:**
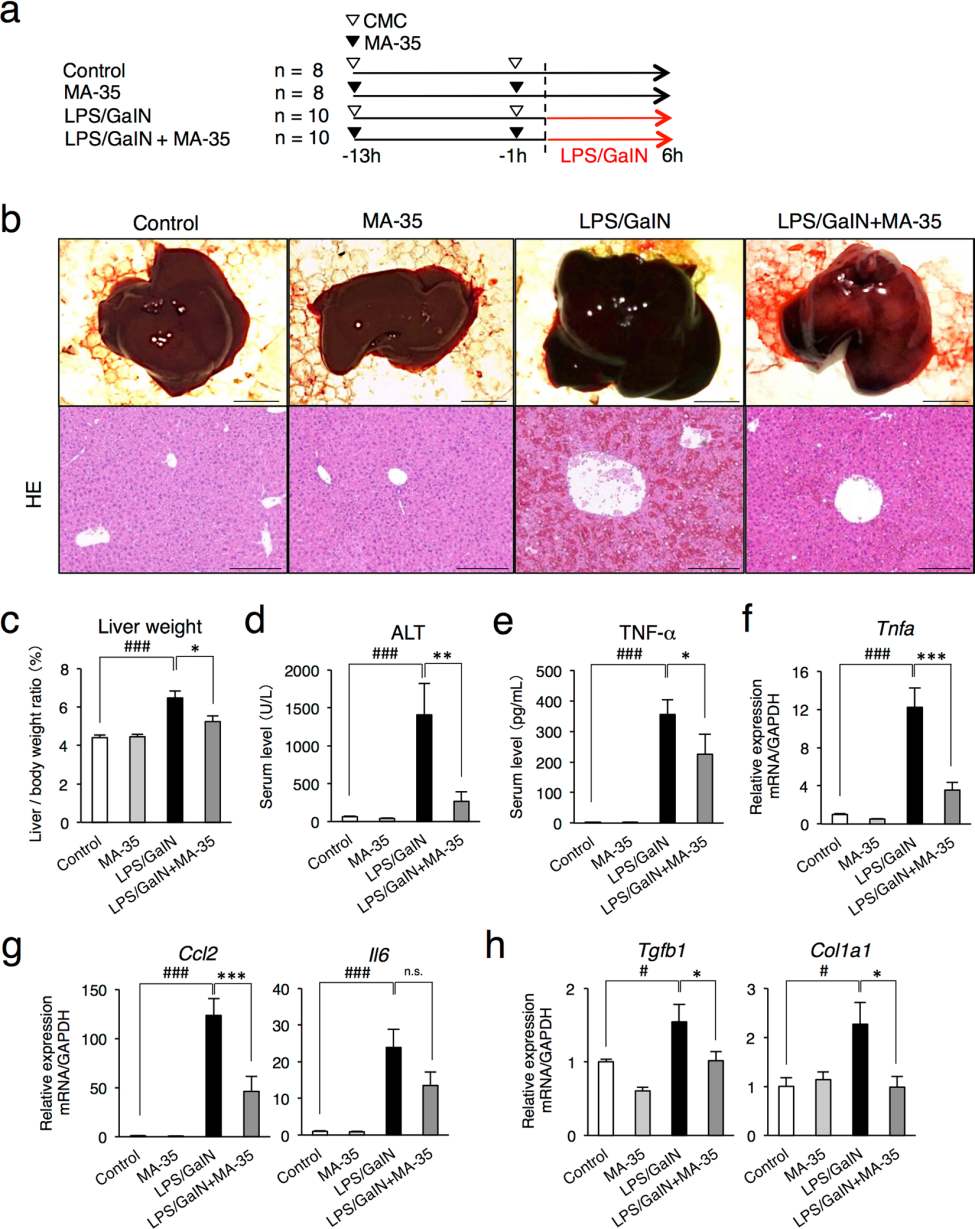
Effect of MA-35 on LPS/GaIN-induced liver injury in mice. (**a**) Experimental design. MA-35 or CMC was orally administrated 13 h and 1 h prior to LPS/D-GaIN injection. All mice were sacrificed at 6 h after the injection. (**b**) Representative gross appearance and HE staining of liver at 6 h after LPS/GaIN injection. Scale bars are 1 cm and 50 μm, respectively. (**c**) Changes in the liver/body weight ratio after LPS/GaIN injection. (**d**) Serum ALT activities were measured at 6 h and (**e**) Serum TNF-α as measured at 1 h after the LPS/GaIN injection. The hepatic mRNA levels of (**f**) *Tnfa*, (**g**) *Ccl2*, *Il6* and (**h**) *Tgfb*_*1*_, *Col1a1* were measured by real-time PCR. ^#^ *p* < 0.05, ^###^ *p* < 0.001 v.s. Control, *p < 0.05, **p < 0.01, ***p < 0.001 v.s. LPS/GaIN.

To examine the anti-TNF-α effect of MA-35 *in vivo*, we next examined the effect in the LPS/D-galactosamine (D-GalN) induced-fulminant hepatitis mice model, in which TNF-α plays a main pathway in the pathogenic condition^16^. As in Fig. 3a, MA-35 was orally administered prior to LPS/D-GalN injection and the mice were sacrificed 6 h after the injection. In the LPS/D-GalN alone-injected mice, the liver was enlarged and showed severe hemorrhaging (Fig. 3b and c). The serum ALT level in the LPS/D-GalN mice was remarkably elevated, demonstrating severe liver injury (Fig. 3d). In this condition, pretreatment of MA-35 significantly attenuated the liver enlargement and histological liver damage induced by LPS/D-GalN (Fig. 3b and c). The elevated serum ALT level was also reduced by MA-35 (Fig. 3d). In addition, LPS/D-GalN markedly increased serum TNF-α level and the hepatic TNF-α mRNA level, which were significantly decreased by MA-35 treatment (Fig. 3e and f). As well as increase of TNF-α, LPS/D-GalN injection was concurrently increased other inflammatory cytokines such as MCP-1 and IL-6 (Fig. 3g). MA-35 significantly reduced the elevated MCP-1 mRNA expression and also tended to reduce the IL-6 mRNA level (Fig. 3g). These results indicate that MA-35 showed the inhibiting effect in the TNF-α-mediated inflammatory response also *in vivo*, which ameliorated the hepatic injury in the mice model.

In the LPS/D-GalN hepatic injury model, TGF-β_1_ mRNA level is reported to be increased^16^. Our analysis also showed that the TGF-β_1_ mRNA expression in the liver was elevated by the LPS/D-GalN. This elevation of hepatic TGF-β_1_ was significantly reduced by MA-35 treatment (Fig. 3h). The expression level of collagen I (*Col1a1*) was also increased by the LPS/D-GalN, and this increase was significantly suppressed by MA-35 (Fig. 3h). As in the case of the TNF-α positive feedback loop, the induced-TGF-β_1_ further activated the TGF-β_1_ signaling and enhanced the TGF-β_1_ expression and the expression of other fibrotic genes such as collagen I. Thus, our findings suggest that MA-35 exerted an anti-TGF-β_1_ effect in addition to the anti-TNF-α effect. Since the TGF-β_1_ signaling is a major driver of fibrosis, we next focused on the effect of potential anti-TGF-β_1_ effect of MA-35.

### MA-35 suppressed TGF-β_1_-induced ECM expression by inhibiting Smad3 phosphorylation

To examine the anti-TGF-β_1_ effect of MA-35 and its mechanism, we investigated the TGF-β_1_ signal pathway. TGF-β_1_ activates Smad3 phosphorylation, which accelerates the translocation of the Smad2/3-Smad4 heterotrimer complex to the nucleus and facilitates the transcription of TGF-β_1_ target genes^5^. This TGF-β_1_ induced-Smad pathway plays a crucial role in the progression of fibrosis^5^.

**Figure 4:**
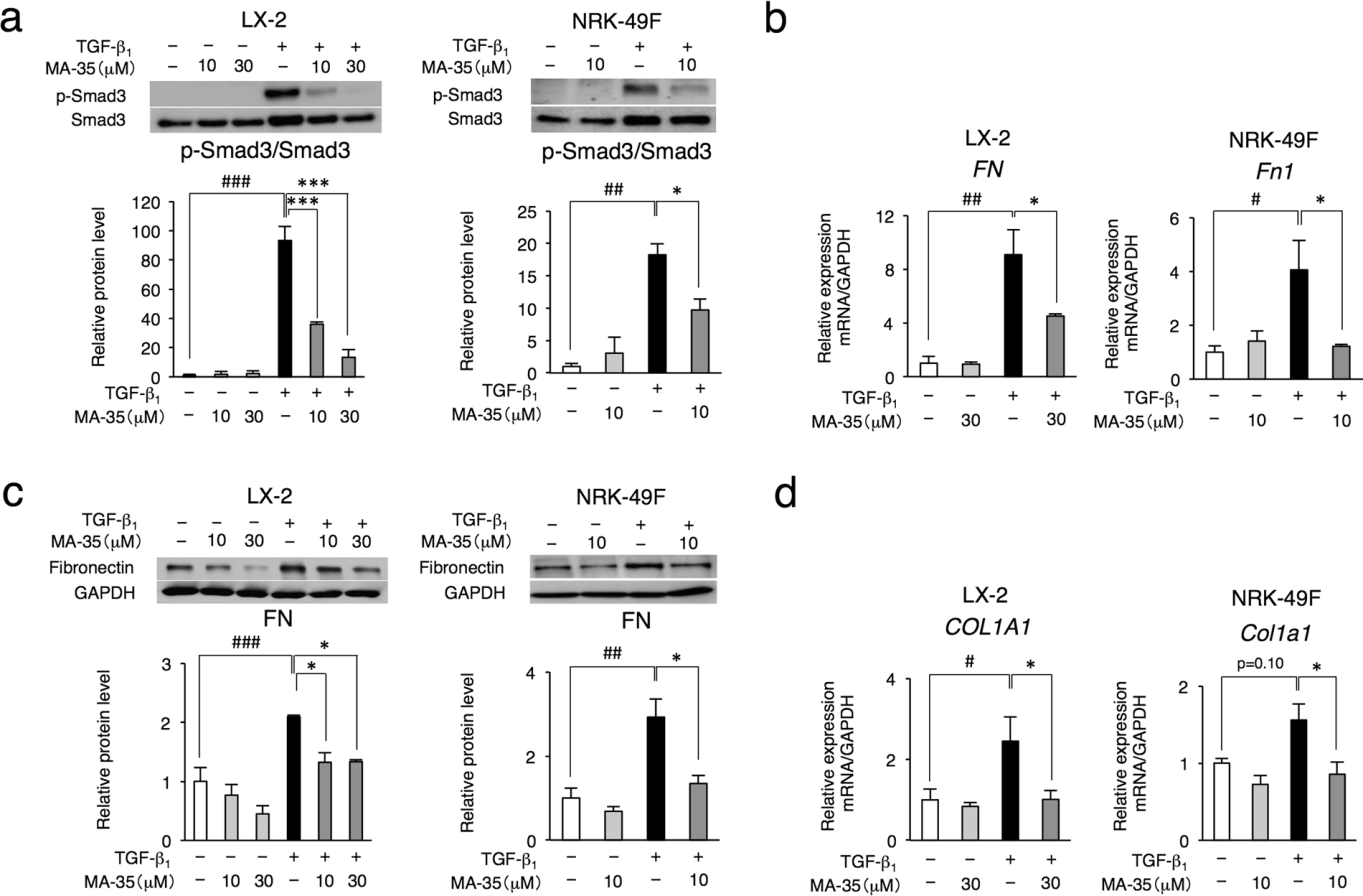
Effect of MA-35 on TGF-β_1_-induced Smad3 phosphorylation and fibrotic gene expression in LX-2 cells and NRK-49F cells. Serum-starved LX-2 and NRK-49F cells were treated with or without TGF-β_1_ (5 ng/mL and 2 ng/mL, respectively) in the presence or absence of MA-35 for (**a**) 30 min, (**b right**) (**d right**) 12 h, (**b left**) (**d left**) 24 h and (**c**) 48 h. (**a**) Expression levels of p-Smad3 were quantified by densitometry and normalized with Smad3. Full-length gel was shown in Supplementary Fig. 6. The mRNA level of (**b**) *FN, Fn1* and (**d**) *COL1A1*, *Col1a1* were measured by real-time PCR. (**c**) Expression levels of fibronectin were quantified by densitometry and normalized with GAPDH. Full-length gel was shown in Supplementary Fig. 7. ^#^p < 0.05, ^##^ *p* < 0.01, ^###^ *p* < 0.001 v.s. Control, *p < 0.05, ***p < 0.001 v.s. TGF-β_1_.

We examined the effect of MA-35 on TGF-β_1_/Smad3 signaling using LX-2 cells and rat kidney interstitial NRK-49F fibroblasts, which are representative cell lines for examining TGF-β_1_-mediated fibrosis. MA-35 alone had no effect on Smad3 phosphorylation in either LX-2 or NRK-49F cells (Fig. 4a). After TGF-β_1_ stimulation, the phosphorylation of Smad3 peaked at 30 min in both cell types (Supplementary Fig. 5a and b). Under this condition, TGF-β_1_ markedly increased Smad3 phosphorylation, and this upregulated Smad3 phosphorylation was significantly inhibited by MA-35 in both LX-2 and NRK-49F cells (Fig. 4a and Supplementary Fig. 6).

Because TGF-β_1_ increases ECM proteins such as fibronectin and collagen I in these cell lines^5^, we next examined the effect of MA-35 on TGF-β_1_-mediated fibronectin at both the mRNA and protein levels. In the basal condition, MA-35 had no effect on the mRNA level of the fibronectin protein in either LX-2 or NRK-49F cells (Fig. 4b). After TGF-β_1_ stimulation, the mRNA expression level of fibronectin was increased and its elevation was significantly inhibited by MA-35 in both LX-2 and NRK-49F cells (Fig. 4b). The fibronectin protein level was also increased by TGF-β_1_ stimulation and its elevation was also inhibited by MA-35 in both cells (Fig. 4c and Supplementary Fig. 7). Similarly, the increased collagen I mRNA level by TGF-β_1_ stimulation was inhibited by MA-35 (Fig. 4d). These data indicate that MA-35 inhibits TGF-β_1_/Smad3 signaling, which reduces the production of ECM.

### MA-35 attenuated interstitial fibrosis in a renal fibrosis model

**Figure 5:**
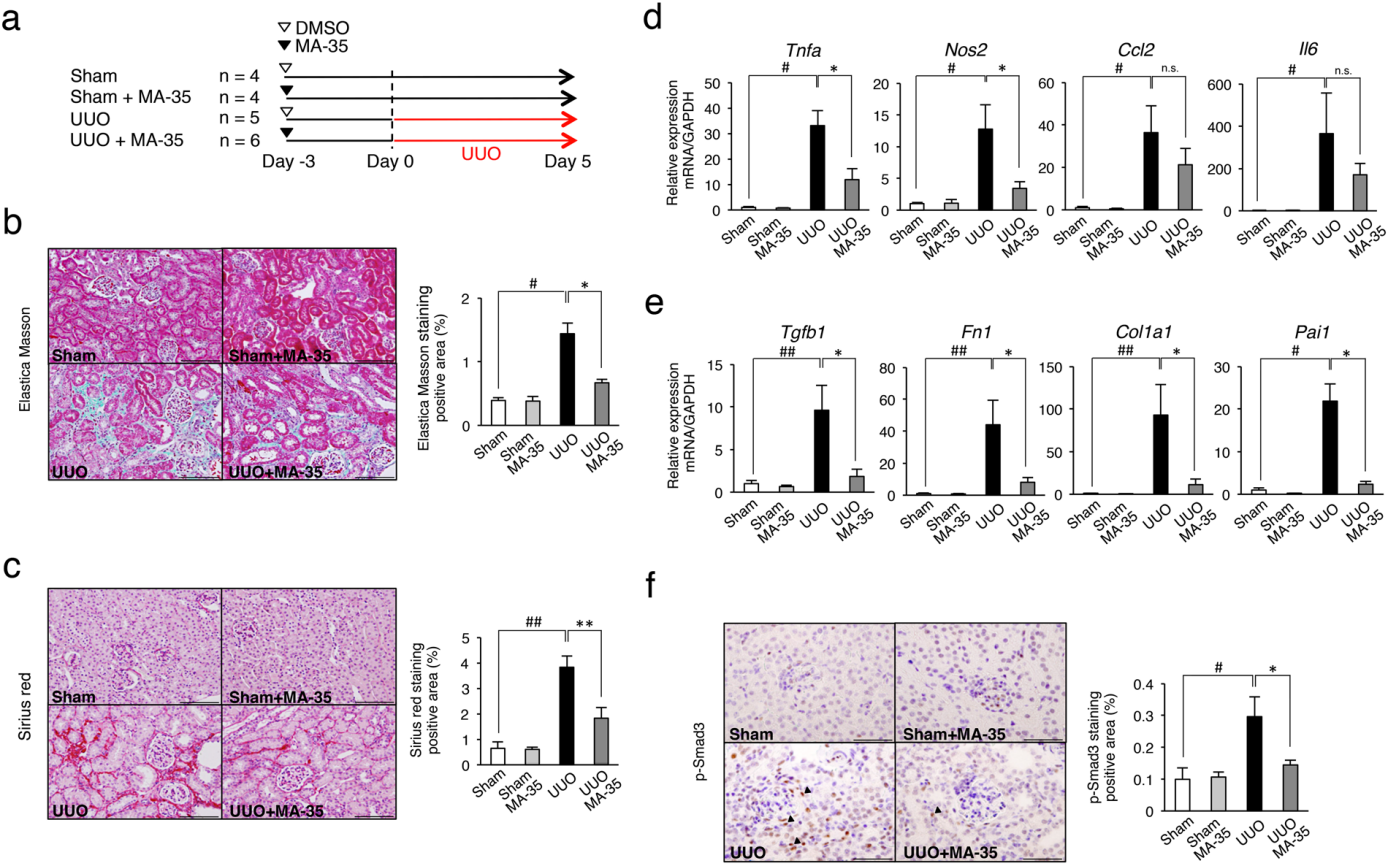
Effect of MA-35 on the renal inflammatory response and fibrosis in the UUO kidney in mice. (**a**) Experimental design. MA-35 or DMSO was administrated intraperitoneally starting from 3 day before UUO until sacrifice 5 days after UUO. Control mice groups received a sham-operation. Representative histological images of (**b**) Elastica Masson and (**c**) Sirius red staining in the kidney. Scale bars, 50 μm. The graph shows the percentage of Elastica Masson-positive and Sirius red-positive areas relative to the whole area. Renal mRNA levels of (**d**) *Tnfa*, *Nos2*, *Ccl2*, *Il6*, (**e**) *Tgfb*_*1*_, *Fn1*, *Col1a1* and *Pai-1* were measured by real-time PCR. (**f**) Representative histological images of p-Smad3 staining in the kidney. Black arrowheads indicate p-Smad3-positive cells. Scale bars, 50 μm. The graph shows the percentage of p-Smad3-positive areas relative to the whole area. ^#^ *p* < 0.05, ^##^ *p* < 0.01 v.s. Sham, *p < 0.05, **p < 0.01 v.s. UUO.

Because our results revealed that MA-35 exerts both anti-TNF-α and anti-TGF-β_1_ effects following the reduction of ECM production, we next investigated the anti-fibrotic effect of MA-35 in a renal fibrosis mice model, unilateral ureteral obstruction (UUO)^17^. Because many previous reports evaluated fibrosis in the early stage of UUO^18, 19^, we also examined the anti-fibrotic effect of MA-35 5 days after the UUO. MA-35 was administered by osmotic pump starting 3 days before the unilateral ureter ligation and the mice were sacrificed 5 days after the ligation (Fig. 5a). As a result, UUO induced severe renal fibrosis indicated by Elastica Masson (Fig. 5b), Sirius red (Fig. 5c) and collagen I staining (Supplementary Fig. 8), which reveals collagen deposition. In this condition, MA-35 significantly decreased the Elastica Masson-positive (Fig. 5b), the Sirius red-positive (Fig. 5c) and collagen I-positive (Supplementary Fig. 8) areas, indicating the attenuation of the progression of renal fibrosis.

In the UUO kidney, the inflammatory genes expression including the TNF-α, iNOS, MCP-1 and IL-6 were elevated (Fig. 5d). The elevated TNF-α and iNOS mRNA levels were significantly reduced by MA-35 treatment (Fig. 5d). MA-35 also tended to suppress the mRNA expression level of MCP-1 and IL-6 (Fig. 5d). We also examined the expression levels of fibrotic genes in the UUO kidney. The expression levels of TGF-β_1_ (*Tgfb1*), fibronectin (*Fn1*), collagen I (*Col1a1*) and plasminogen activator inhibitor-1 (PAI-1) (*Pai1*) were increased in the UUO kidney, and this increase was significantly suppressed by MA-35 (Fig. 5e). In addition, UUO kidney showed an increase in the p-Smad3 level, which was significantly decreased by MA-35 treatment (Fig. 5f). These data indicate that MA-35 attenuated renal fibrosis by reducing inflammatory cytokine expression through TNF-α pathway and fibrotic genes expression through the TGF-β_1_/Smad3 dependent pathway.

### MA-35 modulates TGF-β_1_-mediated histone methylation

**Figure 6:**
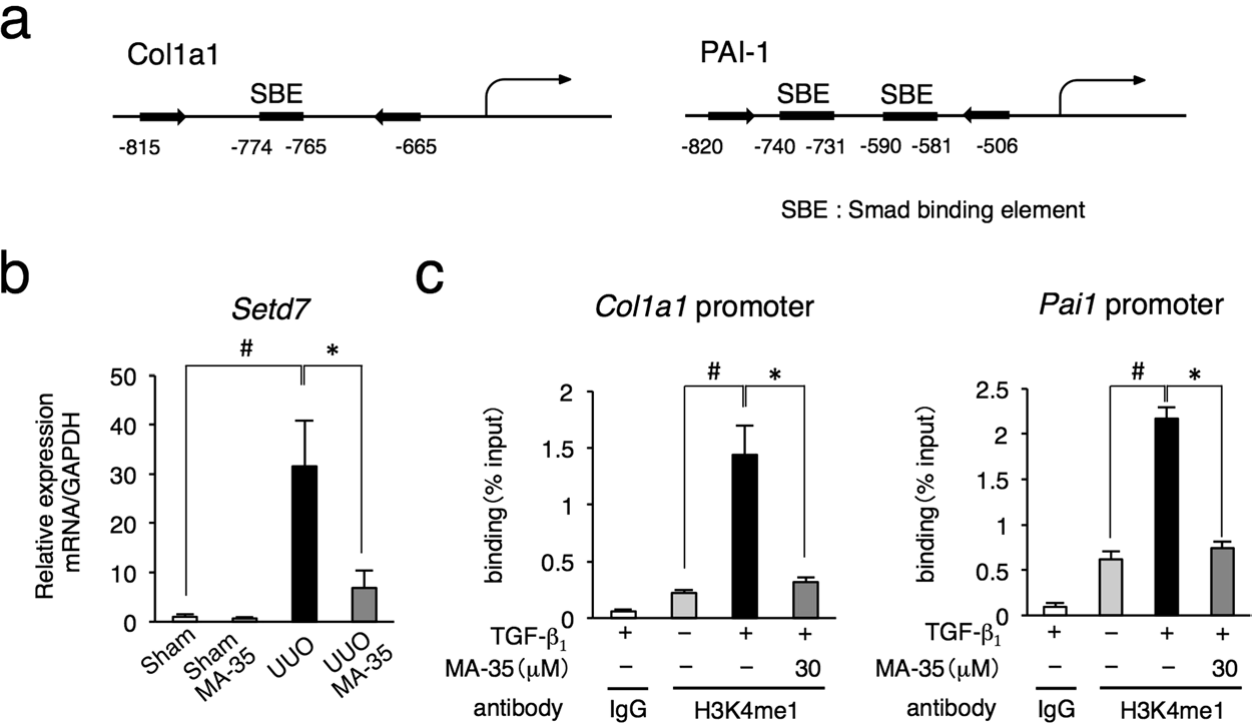
Effect of MA-35 on renal epigenetic modifications, histone methyltransferase, SET7/9 and H3K4me1 both *in vitro* and *in vivo*. (**a**) The map shows the location of *Cola1a* and *PAI-1* gene promoter primer used for ChIP-qPCR. Renal mRNA level of (**b**) *Setd7* was measured by real-time PCR. ^#^ *p* < 0.05 v.s. Sham, *p < 0.05 v.s. UUO. (**c**) ChIP assay analysis of the expression of binding of H3K4me1 protein to the *Col1a1* and *PAI-1* promoters in NRK-52E cells. Immunoprecipitated DNA and input DNA were subjected to qRT-PCR. Rabbit immunoglobulin G (IgG) was used as a negative control. Results were normalized to input DNA. ^#^ *p* < 0.05 v.s. Control, *p < 0.05 v.s. TGF-β_1_.

Recently, it was reported that epigenetic regulations such as histone modifications involved in the progression of fibrosis^6^. Because MA-35 ameliorated fibrosis by inhibiting TGF-β_1_/Smad3 signaling both *in vitro* and *in vivo*, we additionally examined the effect of MA-35 on epigenetic regulations. SET7/9 is an enzyme which methylates histone H3K4, and the resultant H3K4me1 activates collagen I and PAI-1 gene transcription binding of the Smad binding element (SBE)^20^ (Fig. 6a). Previous our report showed that SET7/9 was increased in the UUO model and the inhibition of SET7/9 decreased renal fibrosis^20^.

In the UUO kidney, the mRNA levels of SET7/9 (*Setd7*) was increased and this increase was significantly suppressed by MA-35 (Fig. 6b). To investigate the effect of MA-35 on the H3K4me1 levels in the fibrotic gene promoters, we performed chromatin immunoprecipitation (ChIP) assays for evaluation the binding of the H3K4me1 protein to fibrotic gene promoters using rat kidney tubular epithelial NRK-52E cells. Figure 6a shows locations of *Cola1a* and *PAI-1* gene promoter primer used for ChIP-qPCR. After TGF-β_1_ stimulation, H3K4me1 binding to the collagen I (*Col1a1*) and PAI-1 (*PAI-1*) promoters was upregulated, and this upregulation was significantly inhibited by MA-35 (Fig. 6c). These results suggest that the anti-fibrotic effect of MA-35 may be partly mediated by epigenetic modification.

## Discussion

**Figure 7:**
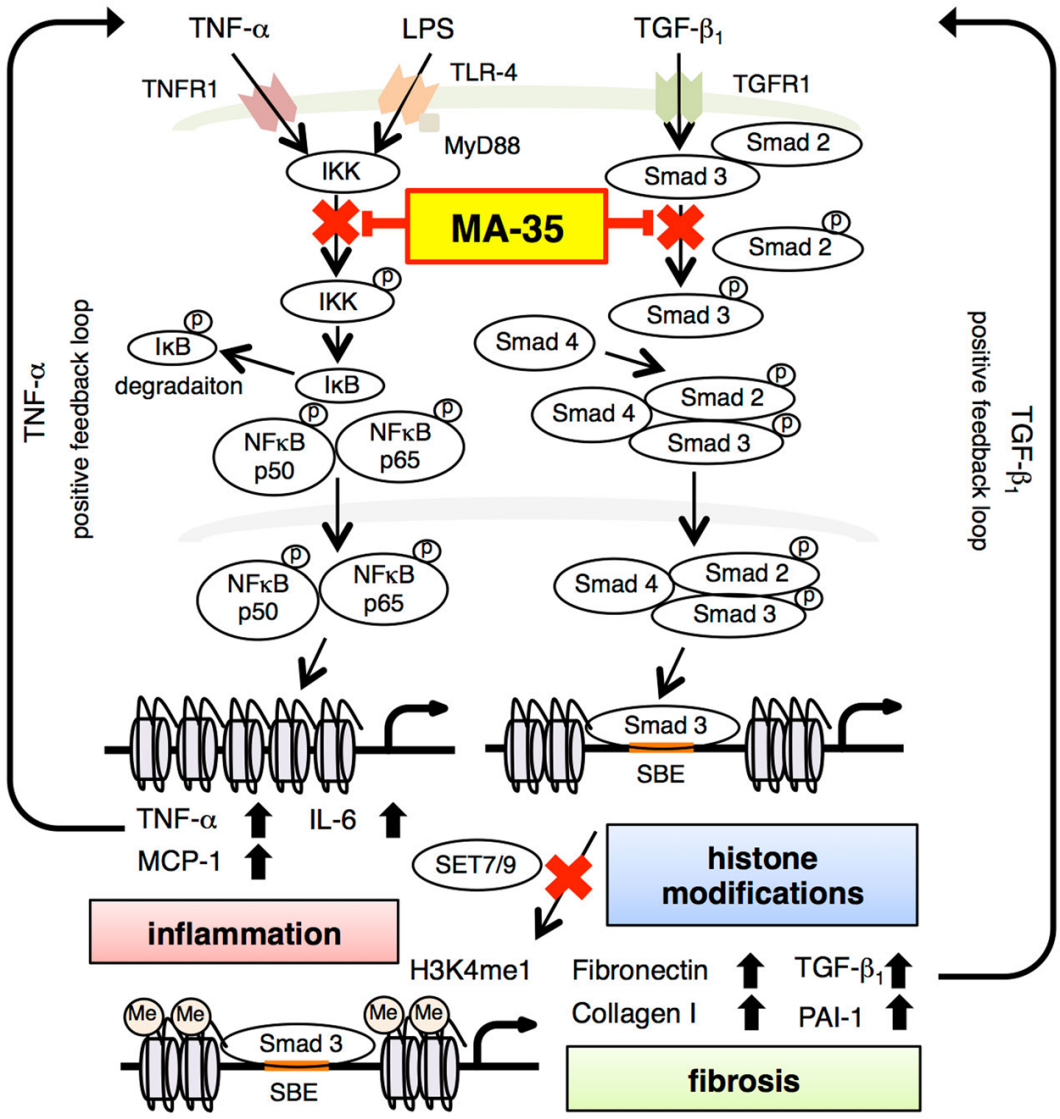
The schema of the expected mechanism for the anti-inflammatory, anti-fibrotic and anti-epigenetic effects of MA-35.

In the present study, we showed that the novel indole derivative compound MA-35 attenuated renal fibrosis by dual inhibition of both the TNF-α and TGF-β_1_ signaling pathways. In addition, MA-35 modulated histone methylation of the H3K4me1 that could be involved in the progression of fibrosis. Since MA-35 have effects on the multiple signaling pathways involved in the fibrosis and may recover epigenetic modification, it thereby would be a potential novel therapeutic drug for anti-fibrotic treatment (Summarized in Fig. 7).

MA-35 exerts an anti-TGF-β_1_ effect by inhibiting Smad3 phosphorylation and reduces the Smad3-driven expression of fibrotic genes such as fibronectin and collagen I. Smad3 is an important downstream mediator of TGF-β_1_ signaling. It is reported that Smad3 signaling was also involved in obesity-related kidney diseases and that a specific Smad3 inhibitor, SIS3, ameliorated the obesity-induced kidney injury^21^. MA-35 also has a Smad3 inhibiting effect and therefore may be effective for such renal diseases as well as renal fibrosis.

Recent studies showed that TGF-β_1_/Smad3 signaling is involved in epigenetic regulation^5^. Epigenetic histone methylation modulates the expression of fibrotic genes such as collagen I and PAI-1, leading to the eventual development of fibrosis^6^. Among many histone modifications, the histone H3K4 monomethylation has been reported to be involved in renal fibrosis^20^. We demonstrated that MA-35 reduced the histone H3K4 modification enzyme SET7/9, resulting in the decrease of H3K4me1 binding to the pro-fibrotic gene promoters. It is not yet clear whether MA-35 directly or indirectly influenced on the condition of SET7/9 and H3K4me1. Nevertheless, whether directly or not, MA-35 eventually reduced the SET7/9 and H3K4me1 levels at the fibrotic gene promoter, leading to an attenuation of renal fibrosis.

In addition to anti-TGF-β_1_ activity, MA-35 exerts an anti-TNF-α effect by inhibiting IKK phosphorylation. Notably, our results suggest that MA-35 is an effective small-molecule inhibitor of TNF-α synthesis in the animal model of fulminant hepatic injury. The effect of MA-35 is another mechanism underlying the anti-TNF-α effect of existing anti-TNF-α biologics. In addition, small molecule compound generally can be produced a relatively lower cost compared to the biologic drugs such as anti-TNF-α biologics^22^. Thus, small molecule compound MA-35 may be expected to offer another choice for treating inflammatory diseases^23, 24^ in which TNF-α is involved. It has been reported that pirfenidone, which is an anti-fibrotic agent used for idiopathic pulmonary fibrosis, also exhibits anti-TNF-α and anti-TGF-β_1_ effect. Although pirfenidone is reported to inhibit TGF-β_1_ synthesis, its detailed mechanism has been yet poorly understood^25^.

MA-35 showed dual blockade of TNF-α and TGF-β_1_ signaling. MA-35 inhibited TNF-α–induced IKK phosphorylation at 30 μM (Fig. 2b). However, MA-35 inhibited TGF-β_1_–induced Smad3 phosphorylation at 10 μM (Fig. 4a). Thus, MA-35 may possess a stronger inhibiting effect on the TGF-β_1_ pathway than on the TNF-α pathway. Since high throughput RNA sequence or other screening techniques were not performed in the present study, we did not evaluate the comprehensive effect of MA-35 apart from its effects on TNF-α, TGF-β_1_, and its epigenetic modification. Thus, MA-35 might have other effects than the three mechanisms that we described, which may be involved in the amelioration of the liver injury and renal fibrosis in the animal model. In addition, high throughput experiments such as microarray or signal transduction pathway search should be needed to further clarify the functional mechanism of MA-35.

Indole is an aromatic heterocyclic organic compound. Indole derivatives have been reported to show various biological activities^26^. We previously showed that another indole compound MA-5 increased cellular ATP level, rescued mitochondrial disease patient fibroblasts, and prolonged the life span of the mitochondrial disease model mouse known as the Mitomouse^8, 9, 27^. Thus, small modifications to indole backbone can offer different biological actions. Our findings show new direction of drug discovery.

In conclusion, MA-35 reduces the inflammatory response by inhibiting TNF-α/IKK signaling and prevents renal fibrosis by inhibiting TGF-β_1_/Smad3 signaling. In addition, MA-35 may aid recovery from epigenetic modification. MA-35 affects multiple cell signaling pathways and epigenetic modifications, and thus could be a potential novel therapeutic drug candidate for renal fibrosis and inflammatory diseases.

## Methods

### Cell culture and treatments

Human hepatoma Hep3B cells (ATCC, #HB-8064) were cultured in Roswell Park Memorial Institute (RPMI) 1640 medium containing 10% fetal bovine serum (FBS), 100 units/mL penicillin and 100 μg/mL streptomycin. Human hepatic stellate cell line LX-2 cells were kindly provided by Prof. Friedman of the Mount Sinai Medical School, New York. LX-2 cells and Murine TNF-α sensitive L929 fibrosarcoma cells (RCB, #1422) were cultured in DMEM containing 10% FBS, 100 units/mL penicillin and 100 μg/mL streptomycin. Normal rat kidney interstitial fibroblasts (NRK-49F) (ATCC, #CRL-1570) and normal rat kidney tubular epithelial cells (NRK-52E) (ECACC, #EC87012902-G0) were cultured in DMEM containing 5% FBS, 100 units/mL penicillin and 100 μg/mL streptomycin. Each cell type was incubated in serum-free medium for 24 h prior to each stimulation. After pretreatment with MA-35 at a dose of 10 μM or 30 μM for 60 min, recombinant human TNF-α (10 ng/mL, PeproTec), LPS (100 ng/mL, Escherichia coli O111: B4, Sigma Aldrich) or recombinant human TGF-β_1_ (2 ng/mL or 5 ng/mL, PeproTec) was added to the culture for indicated periods of time. Samples were subjected to quantitative PCR or Western blot (n = 3).

### Cell viability assay

LX-2 cells were treated with indicated concentrations of MA-35 in serum-free DMEM for 24 h. Cell viability was measured using Cell Count Reagent SF (Nacalai Tesque) (n = 6). L929 cells were treated with TNF-α (0.03 ng/mL) and actinomycin D (10 μg/mL, Nacalai Tesque) for 20 h in the presence or absence of MA-35 at a dose of 10 μM. Cell viability was measured using a CellTiter 96 Aqueous non-radioactive cell proliferation kit (Promega) (n = 8).

### Animals

All animal experiments were approved by the Tohoku University Animal Care Committee. The experimental protocols and animal care were performed according to the guidelines for animal experiments of Tohoku University. C57BL/6 male mice aged 8–10 weeks were purchased from CLEA Japan, Inc. Acute hepatitis was induced by intraperitoneal injection of LPS (5 μg/kg body weight) and D-GaIN (500 mg/kg body weight, Wako) dissolved in phosphate-buffered saline (PBS). Mice were randomly divided into four groups: calboxymethylcellulose (CMC) (n = 8), MA-35 (n = 8), LPS/D-GaIN (n = 10) and LPS/D-GaIN+MA-35 (n = 10). MA-35 or CMC was orally administered 13 h and 1 h prior to LPS/D-GaIN. Mice were sacrificed to assess the liver damage at 6 h after the LPS/D-GaIN injection.

UUO was performed by ligating the left ureter with 4–0 silk at two points and cutting between the ligatures. Mice were randomly divided into four groups: Sham operation+DMSO (n = 4), Sham operation+MA-35 (n = 4), UUO+DMSO (n = 5) and UUO+MA-35 (n = 6). MA-35 at a dose of 80 mg/kg or DMSO was injected by an intraperitoneally-implanted micro-osmotic pump (Alzet) from 3 days before UUO until they were sacrificed at 5 days after UUO.

### Histrogical analysis and immunohistochemical staining

The livers and kidneys were fixed in 10% buffered formalin and embedded in paraffin. Liver sections were stained with hematoxylin and eosin (HE). Kidney sections were stained with Elastica Masson, Sirius red, collagen I and p-Smad3 antibody. The fibrotic area and p-Smad3-positive area were quantified by measurement at ×200 and ×400 magnification, respectively in 10 randomly non-overlapping fields using the National Institutes of Health Image J software.

### Epo measurement

Hep3B cells were incubated under normoxic condition for 24 h. Cells were further treated with recombinant human TNF-α (220 U/mL) and our 41 chemical compounds (MA-1 to MA-41) in the culture medium under hypoxic conditions (1% O_2_) for 48 h. Human erythropoietin protein was measured using a Human Erythropoietin Platinum ELISA kit (eBioscience). Mice were injected intravenously with MA-35 at a dose of 40 mg/kg or DMSO (n = 3). The murine Epo levels in the plasma were measured at 4 h after injection using a Quantikine ELISA kit (R&D Systems).

### ELISA

The serum ALT level was measured with Fuji DRI-CHEM SLIDE GPT/ALT-PIII (Fujifilm). Murine TNF-α level was measured using a TNF-α ELISA kit (R&D system). All were performed according to the manufacturer’s instructions.

### Reverse transcription and Quantitative PCR

Total RNA was extracted using TriPure Isolation Reagent (Roche) according to the manufacturer’s instructions. cDNA was synthesized using a transcriptor first-strand cDNA synthesis kit (Roche). Quantitative real-time PCR was performed using the Taqman Gene Expression Assay (Applied Biosystems), according to the manufacturer’s instructions using a StepOnePlus^TM^ Real Time PCR System (Applied Biosystems). The primers used are listed in Supplementary Table 1a. The cycle threshold (Ct) was calculated using the CT method. Relative mRNA expression was normalized with GAPDH.

### Western blot

Equal amounts of protein were separated by SDS-PAGE on 10% gel. Proteins were transferred onto a polyvinylidene difluoride membrane. The membrane was incubated overnight with primary antibodies against p-IKK, IKK, p-p38, p38, p-ERK1/2, ERK1/2, p-JNK, JNK, p-Smad3, Smad3 (all from Cell Signaling Technology) and, fibronectin (Santa Cruz Biotechnology), incubated for 1 h with HRP-conjugated secondary antibodies (PIERCE). Protein bands were detected using the enhanced chemiluminescent plus system. Bands intensities were analyzed by NIH Image J software.

### ChIP assay

ChIP assay of *Col1a1* and *PAI-1* was performed using a ChIP Assay Kit (EMD Millipore) according to the manufacturer’s protocol. DNA was purified using the QIAquick PCR Purification Kit (Qiagen). Antibody used is anti-H3K4me1 (Cell Signaling Technology). Purified DNA samples were subjected to qRT-PCR. Results were normalized to input DNA (n = 5). The primers used in this study are listed in Supplementary Table 1b.

### Statistical analyses

All data were expressed as the mean ± SEM. Statistical analyses were performed with one-way ANOVA, Tukey’s test and Kruskal-Wallis test using JMP version 11 (SAS Institute Inc). *P* < 0.05 was considered to be statistically significant.

## Electronic supplementary material

Supplementary Information
