# Supplementary material for: A novel indole compound MA-35 attenuates renal fibrosis by inhibiting both TNF-α and TGF-β1 pathways
Source: Sci Rep. 2017 May 15;7:1884. doi: 10.1038/s41598-017-01702-7 (PMC5432497; doi:10.1038/s41598-017-01702-7)
Supplement: Supplementary file 1 — Supplementary Information [file 41598_2017_1702_MOESM1_ESM.pdf]

Supplementary Information

A novel indole compound MA-35 attenuates renal fibrosis by inhibiting both TNF-α and TGF-β_1 pathways

Hisato Shima[1], Kensuke Sasaki[2], Takehiro Suzuki[1,3], Chikahisa Mukawa[4], Ten Obara[5], Yuki Oba[1], Akihiro Matsuo[1], Takayasu Kobayashi[6], Eikan Mishima[1], Shun Watanabe[1], Yasutoshi Akiyama[1], Koichi Kikuchi[1,7], Tetsuro Matsuhashi[8], Yoshitsugu Oikawa[8], Fumika Nanto[1,3], Yukako Akiyama[1], Hsin-Jung Ho[1,3], Chitose Suzuki[1], Daisuke Saigusa[9], Atsushi Masamune[10], Yoshihisa Tomioka[4], Takao Masaki[2], Sadayoshi Ito[1], Ken-ichiro Hayashi[11] and Takaaki Abe[1,3,7]

1. Division of Nephrology, Endocrinology, and Vascular Medicine, Tohoku University Graduate School of Medicine, Sendai 980-8574, Japan
2. Department of Nephrology, Hiroshima University Hospital, Hiroshima 734-8551, Japan
3. Department of Medical Science, Tohoku University Graduate School of Biomedical Engineering, Sendai 980-8574, Japan
4. Laboratory of Oncology, Pharmacy Practice and Sciences, Tohoku University Graduate School of Pharmaceutical Sciences, Sendai 980-8578, Japan
5. Department of Biology, Indiana University Purdue University Indianapolis, Indianapolis, IN 46202
6. Center for Gene Research, Tohoku University, Sendai 980-8575, Japan
7. Department of Clinical Biology and Hormonal Regulation, Tohoku University Graduate School of Medicine, Sendai 980-8574, Japan
8. Division of Pediatrics, Tohoku University Graduate School of Medicine, Sendai, Sendai 980-8574, Japan
9. Department of Integrative Genomics, Tohoku Medical Megabank Organization, Tohoku University, Sendai 980-8574, Japan
10. Division of Gastroenterology, Tohoku University Graduate School of Medicine, Sendai 980-8574, Japan
11. Department of Biochemistry, Okayama University of Science, Okayama 700-0005, Japan

¶To whom correspondence should be addressed:
Takaaki ABE M.D., Ph.D.
e-mail: takaabe@med.tohoku.ac.jp

Supplementary Figure 1

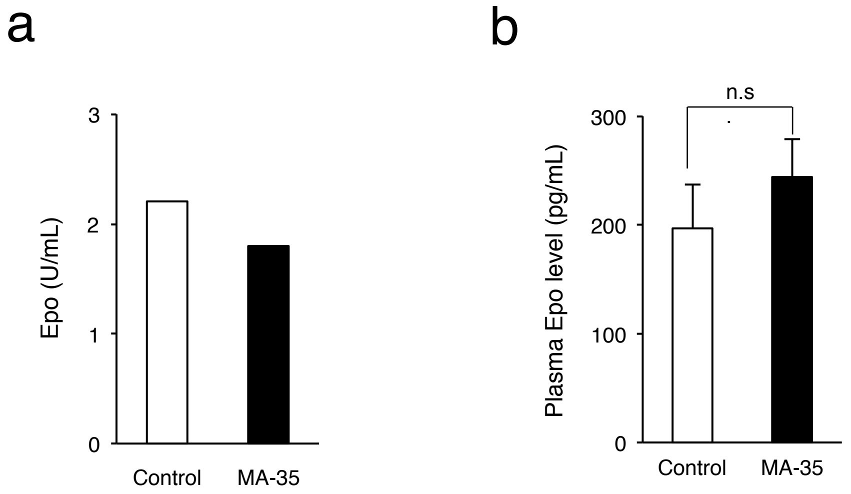

Supplementary Figure 1
(a) Hep3B cells were incubated under normoxic condition for 24 h.
MA-35 alone did not increase Epo level in the culture medium (n=1).
(b) The serum Epo level of mice injected intravenously with MA-35 was not significantly increased (n=3).

Supplementary Figure 2

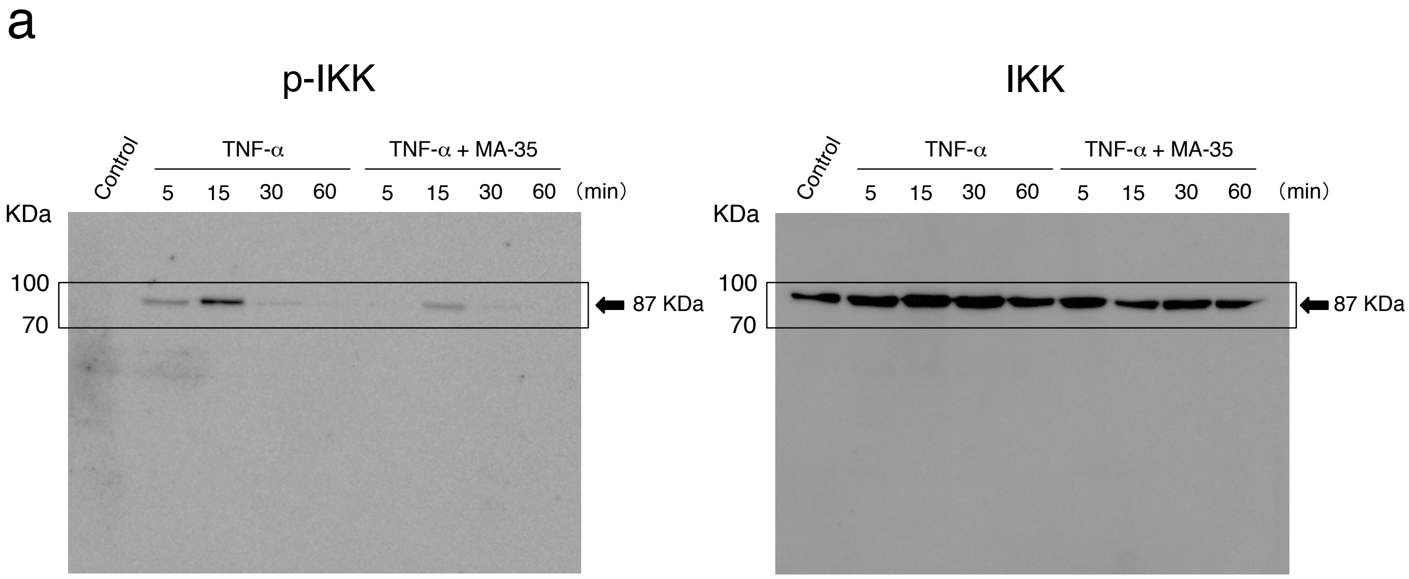

Supplementary Figure 2
Full length blots of Figure 2a.
The time course of TNF-α–induced IKK phosphorylation in LX-2 cells.
MA-35 inhibited TNF-α–induced IKK phosphorylation in a time dependent manner.

Supplementary Figure 3

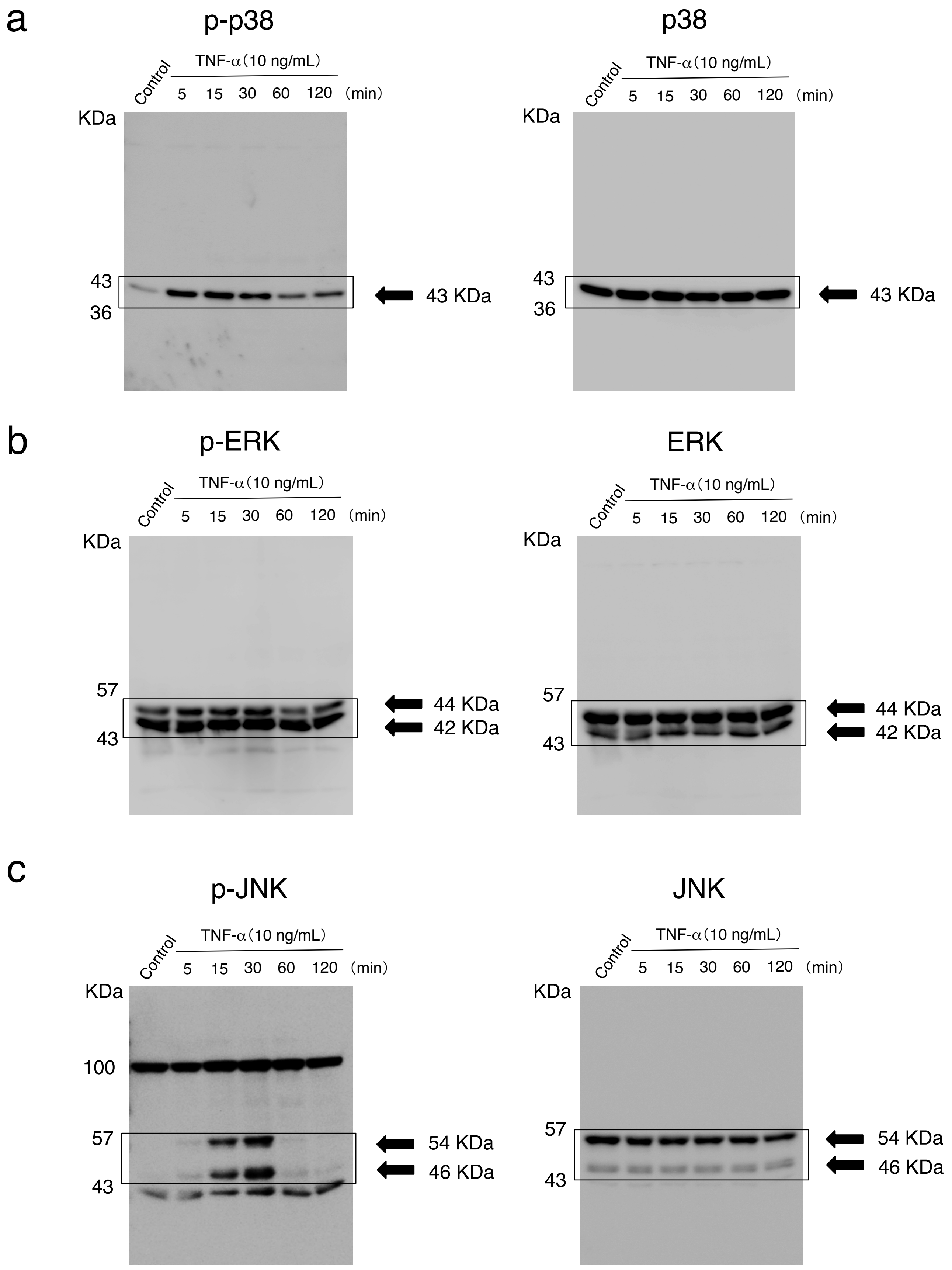

Supplementary Figure 3
The time course of TNF-α–induced (a) p38, (b) ERK and (c) JNK phosphorylation in LX-2 cells.

Supplementary Figure 4

a

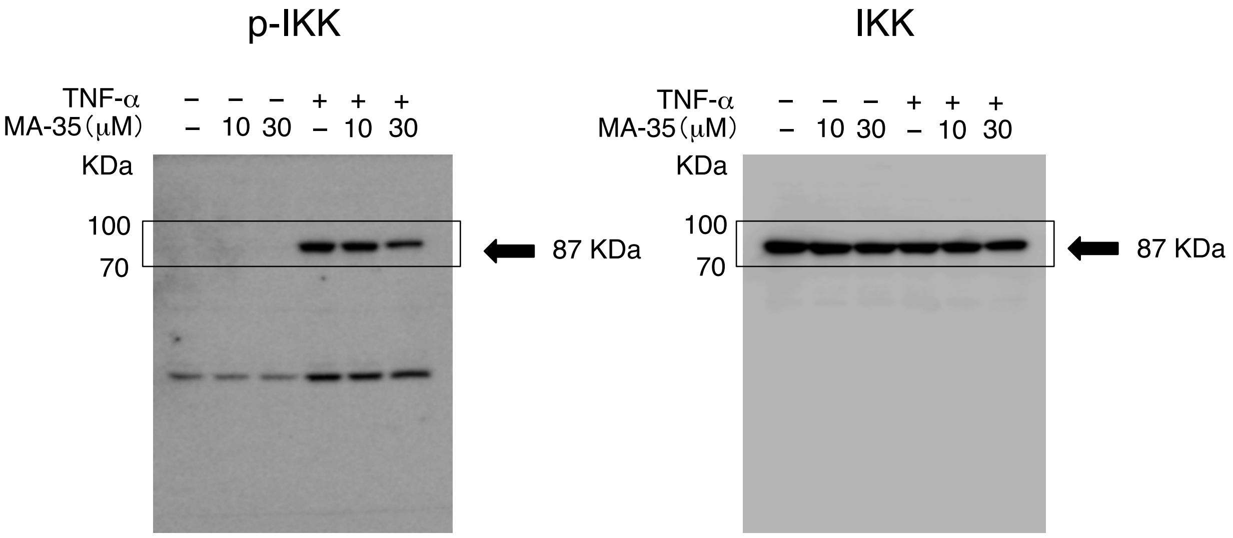

b

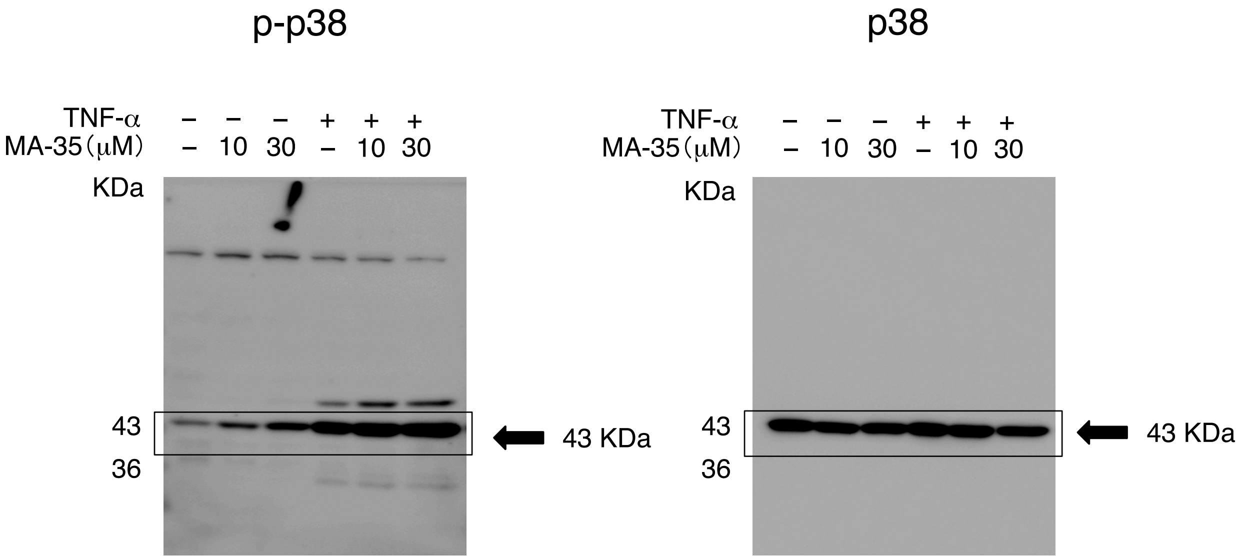

Supplementary Figure 4a
Full length blots of Figure 2b.

Supplementary Figure 4b
Full length blots of Figure 2c.

Supplementary Figure 4（continued）

c

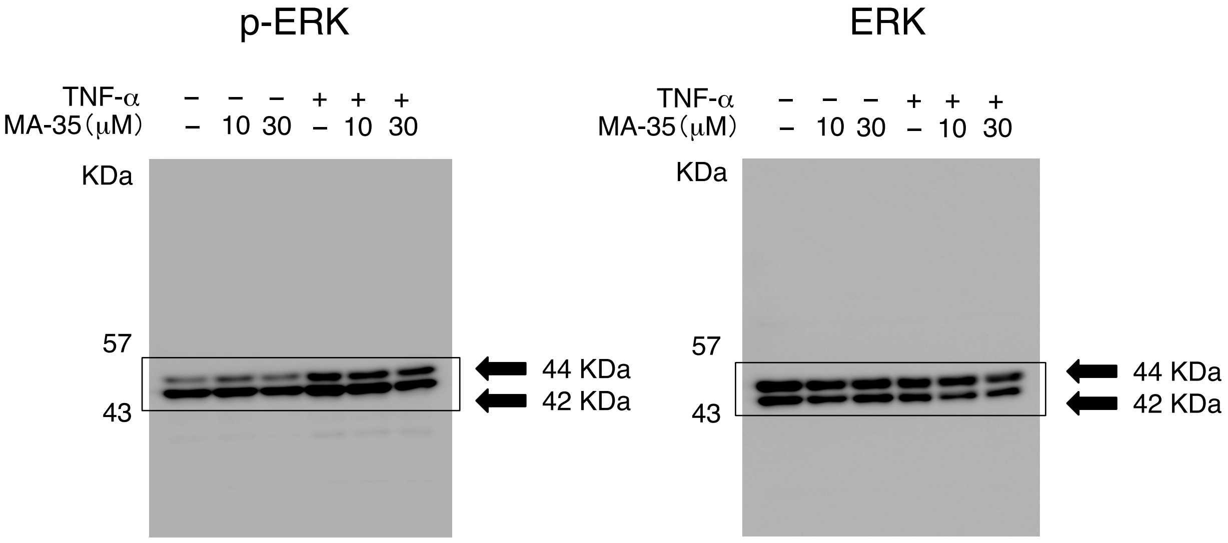

d

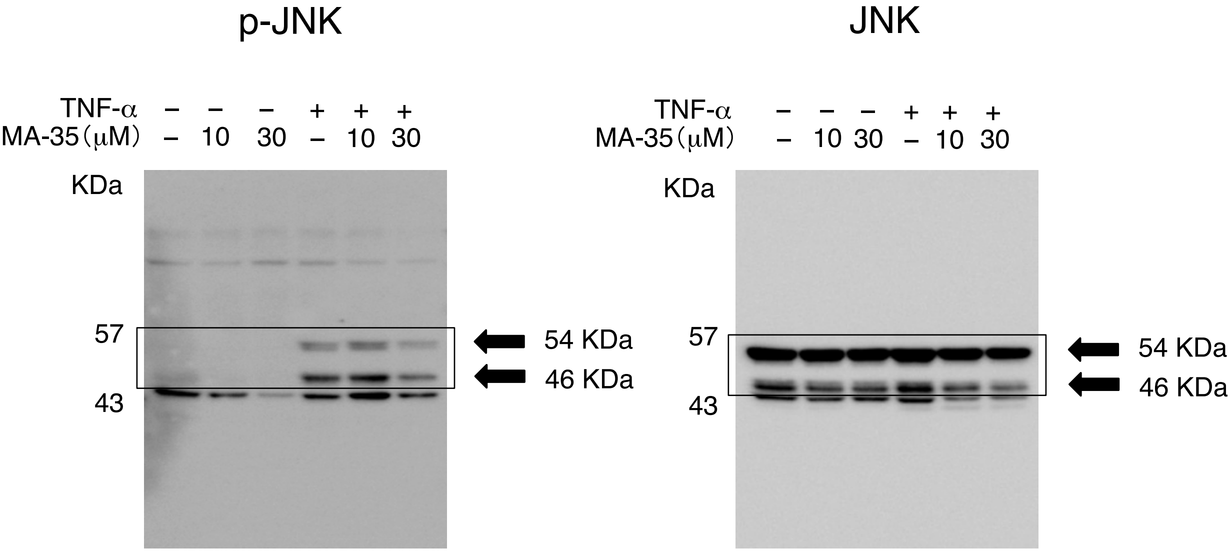

Supplementary Figure 4c
Full length blots of Figure 2c.

Supplementary Figure 4d
Full length blots of Figure 2c.

Supplementary Figure 5

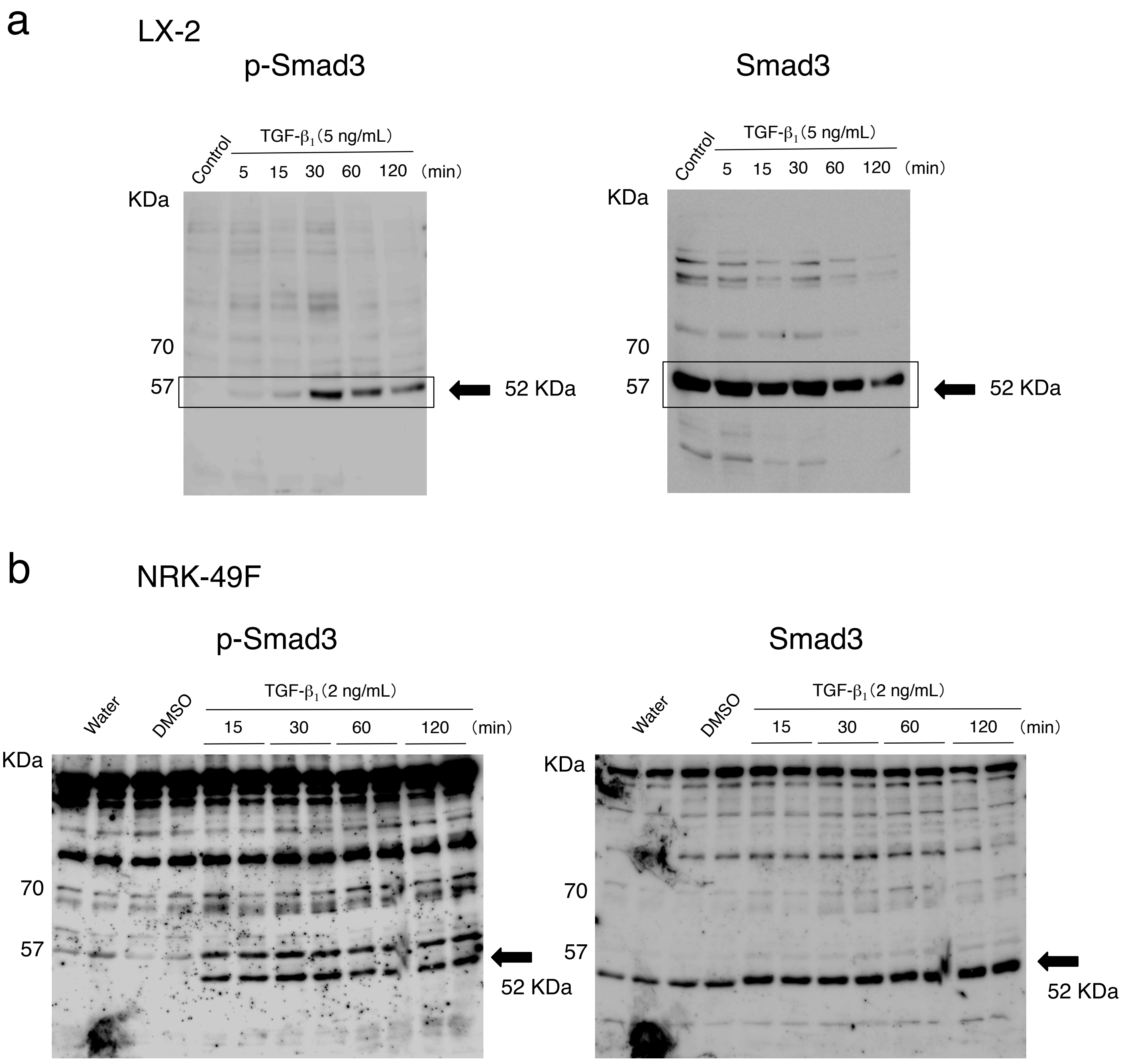

Supplementary Figure 5
The time course of TGF-β_1–induced Smad3 phosphorylation in (a) LX-2 cells and (b) NRK-49F cells.

Supplementary Figure 6

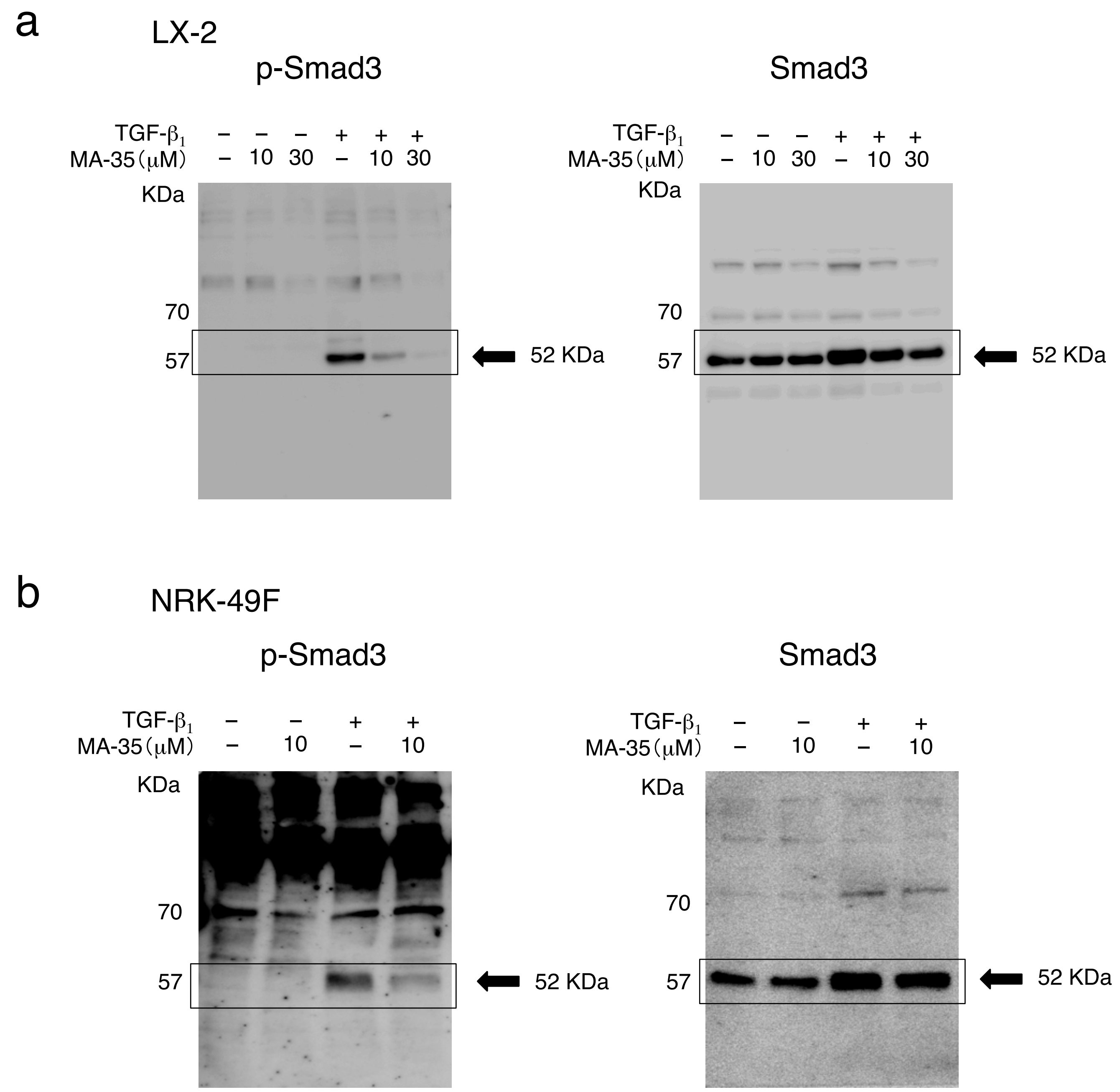

Supplementary Figure 6a, 6b
Full length blots of Figure 4a.

Supplementary Figure 7

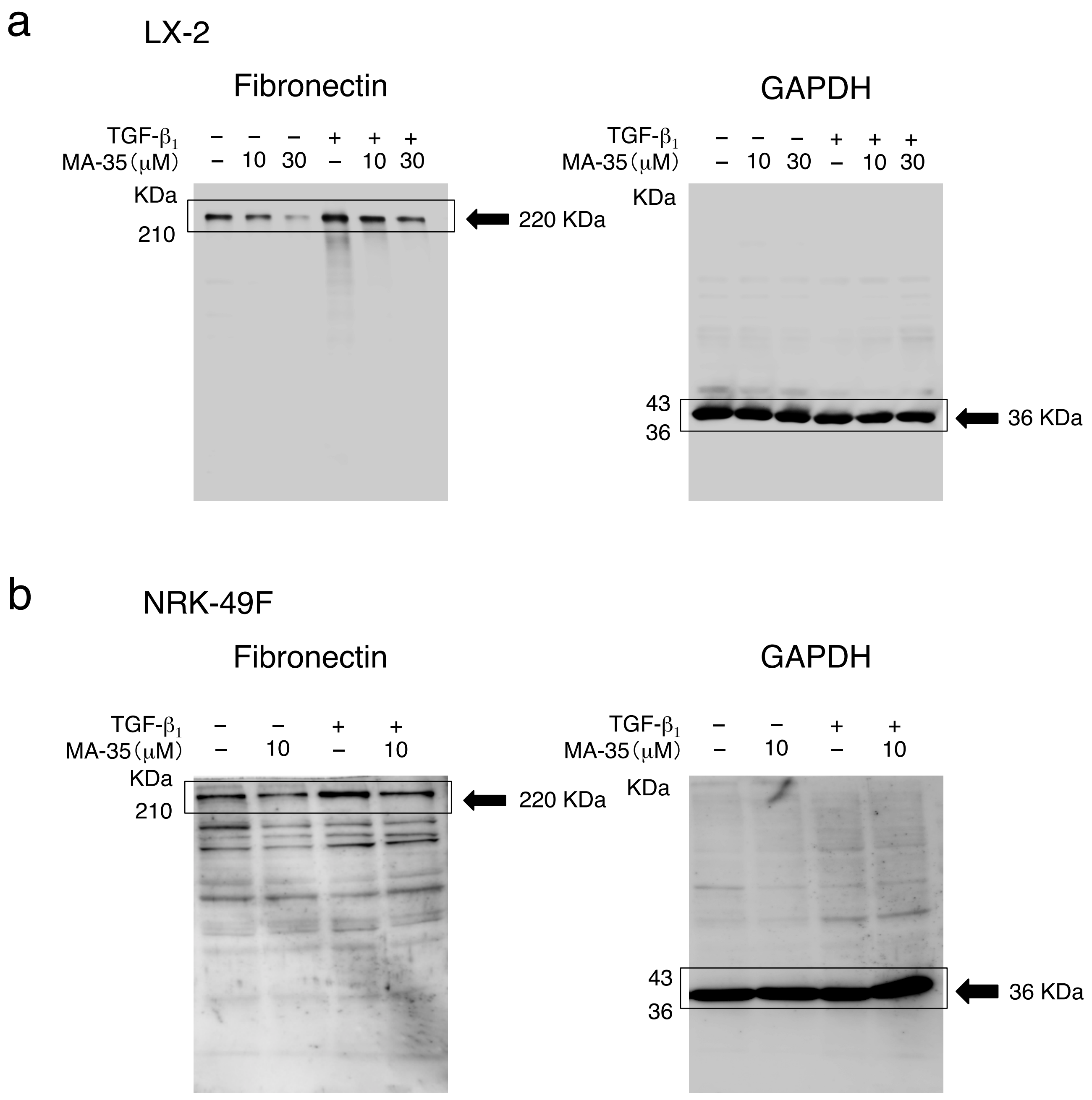

Supplementary Figure 7a, 7b
Full length blots of Figure 4c.

Supplementary Figure 8

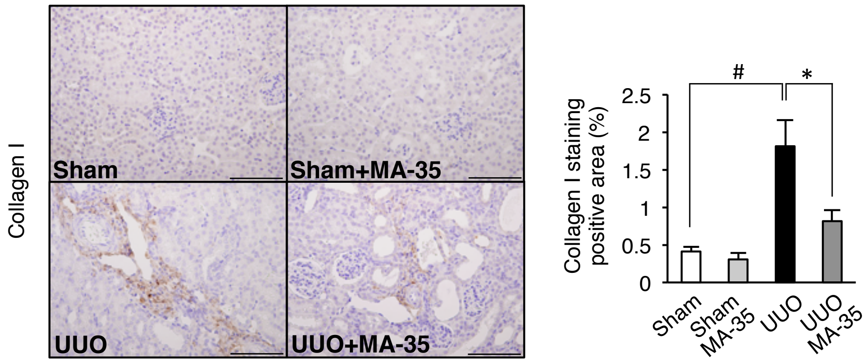

Supplementary Figure 8
Representative histological images of collagen I staining in the kidney.
Scale bars, 50 μm. The graph shows the percentage of collagen I-positive areas relative to the whole area. $^{\#}p < 0.05$ v.s. Sham, $^{*}p < 0.05$ v.s. UUO.

Supplementary Table 1

a

cDNA taqman primers

Gene（Human）	
FN1	Hs00365052_m1
COL1A1	Hs00164004_m1
TNF	Hs01113624_g1
CCL2	Hs00234140_m1
GAPDH	Hs02758991_g1

Gene（Rat）	
Fn1	Rn00569575_m1
Col1a1	Rn01463848_m1
Gapdh	Rn99999916_m1

Gene（Mouse）	
Fn1	Mm01256744_m1
Col1a1	Mm00801666_g1
Tgfb1	Mm01178820_m1
Pai1	Mm00435860_m1
Tnfa	Mm00443260_g1
Nos2	Mm00440485_m1
Ccl2	Mm00441242_m1
Il6	Mm00446190_m1
Setd7	Mm00499823_m1
Gapdh	Mm99999915_m1

b

ChIP primers

Primer	Forward	Reverse
Col1a1	GGCTGGAGAAAGGTGGGTCT	CCCAGGTATGCAGGGTAGGA
PAI-1	gacaatATGTGCCCTGTGATTGtC	AGGCTGCTCTACTGGTCCTTGC
